# 200 kHz Commercial Sonar Systems Generate Lower Frequency Side Lobes Audible to Some Marine Mammals

**DOI:** 10.1371/journal.pone.0095315

**Published:** 2014-04-15

**Authors:** Z. Daniel Deng, Brandon L. Southall, Thomas J. Carlson, Jinshan Xu, Jayson J. Martinez, Mark A. Weiland, John M. Ingraham

**Affiliations:** 1 Energy and Environment Directorate, Pacific Northwest National Laboratory, Richland, Washington, United States of America; 2 Southall Environmental Associates, Aptos, California, United States of America; University of California, Irvine, United States of America

## Abstract

The spectral properties of pulses transmitted by three commercially available 200 kHz echo sounders were measured to assess the possibility that marine mammals might hear sound energy below the center (carrier) frequency that may be generated by transmitting short rectangular pulses. All three sounders were found to generate sound at frequencies below the center frequency and within the hearing range of some marine mammals, e.g. killer whales, false killer whales, beluga whales, Atlantic bottlenose dolphins, harbor porpoises, and others. The frequencies of these sub-harmonic sounds ranged from 90 to 130 kHz. These sounds were likely detectable by the animals over distances up to several hundred meters but were well below potentially harmful levels. The sounds generated by the sounders could potentially affect the behavior of marine mammals within fairly close proximity to the sources and therefore the exclusion of echo sounders from environmental impact analysis based solely on the center frequency output in relation to the range of marine mammal hearing should be reconsidered.

## Introduction

Active acoustic systems transmit sound, usually in short pulses, and receive echoes from targets in the water permitting localization and characterization of targets. Active acoustic systems are ubiquitous in the marine environment, and used convergently by humans and non-human animals, because sound propagates well in water and a very broad range of sensing applications is achievable using sound. Most vessels have one or more echo sounders to aid in navigation [1]. Commercial and recreational fishermen use a wide range of active acoustic systems to locate fish. Various exploration and construction activities use tools that generate and receive sound in configurations that are similar to active acoustic systems [2]. Additionally, militaries use sonar systems for a variety of underwater assessment and communication tasks.

Because active sonar systems sometimes generate intense sounds and many marine animals rely on hearing and sound communication for many critical life functions, there has been considerable interest, concern, and research into the effects of sound on some species [3], [4], [5], [6], [7]. Various configurations of active acoustic systems are being investigated to help understand and reduce potential negative effects of sounds from military sonar systems, maritime construction, seismic exploration, and offshore power production activities. A commonly used approach to reducing the potential impacts of acoustic systems on marine mammals is to move the operating frequency outside the range of functional hearing. While the upper hearing limits vary widely (from a few Hz to perhaps 160 kHz) for fish and marine mammals [3], [8], [9], no marine species are believed to be functionally sensitive to sounds above 200 kHz. Sonars and other active acoustic systems operating at this frequency and higher are generally believed to be inaudible and thus unable to impact marine mammals or other species; such sources have consequently been commonly exempted from environmental permitting requirements for use around protected species. However, while the operating frequency of these sonars is above the hearing range of marine mammals, their operation can generate sound energy outside the specified center frequency that may fall within functional hearing range and be detectable and thus elicit a behavioral reaction or perhaps affect their hearing over small ranges. Researchers at the Pacific Northwest National Laboratory were tasked by the U.S. Department of Energy to develop a system to monitor the presence of marine animals in the presence of a proposed marine hydrokinetic tidal turbine in the Puget Sound [10]. Of primary concern in the Puget Sound was the endangered Southern Resident Killer Whale (*Orcinus Orca*). Initially both passive and active systems were investigated, but the development of the active system had to be discontinued after the National Oceanic and Atmospheric Administration (NOAA) Fisheries expressed concerns that active sonars that operate at 200 kHz may generate sounds that are within the hearing range of killer whales.

The majority of active acoustic systems operate by transmitting short pulses of sound, typically on the order of a millisecond or less in duration. Short transmit pulses are needed to more accurately estimate the location of a target and to distinguish targets that may be located in near proximity to one another. The transmitted pulses also typically have short rise (ring-up) and short fall (ring-down) times relative to the duration of the time the transmit pulse spends at full power. The resulting transmitted pulse is more or less rectangular in shape. Generation of these short, rectangular pulses requires a bandwidth that is inversely proportional to the length of the transmitted pulse. The shorter the pulse duration and the more rapid the ring-up and ring-down times the greater the bandwidth required.

In addition to short rectangular transmit pulses, active acoustic systems also typically have relatively high source levels. High source levels are needed to optimize the effective detection range of the systems, increase the acoustic energy scattered back to the acoustic system receiver by targets with small acoustic cross sections, and permit the use of short pulse lengths to achieve high spatial resolution for isolation of single targets and to more accurately estimate their location. A consequence of high source levels is that, while down several orders of magnitude from the sound level at the frequency of operation, the level of sound at frequencies well away from the frequency of operation may be above the hearing threshold for some marine mammals. Such sound peripheral to the operating frequency are necessary to mechanically achieve the rapid rise and fall times that define the rectangular pulse's characteristics for most active acoustic systems. These sounds occur at the sub-harmonic frequency of the sounders center operating frequency. The sub-harmonic frequency of a sounder with a center frequency of 200 kHz can fall within the hearing range of killer whales, which are expected to have an upper frequency limit of at least 120 kHz based on data from other odontocetes [3], [11] (available audiograms only extend up to 100 kHz and were obtained using electrophysiological methods [12]).

Several such systems were evaluated as an element of design of a mixed passive and active acoustic system for detection of killer whales near tidal power turbines in Admiralty Inlet, WA. In this analysis we consider the level of sound generated at frequencies within the hearing range of killer whales by active acoustic systems that were designed and specified to operate near a center frequency of 200 kHz.

## Methods

Three commercial active sonar systems were evaluated: SM2000 multibeam imaging sonar (hereafter referred to as Kongsberg; Kongsberg Mesotech Ltd., Vancouver, British Columbia, Canada), DT-X Digital Scientific Echosounder (hereafter referred to as BioSonics; BioSonics, Inc., Seattle, Washington), and Model 965 multibeam imaging sonar (hereafter referred to as Imagenex; Imagenex Technology Corp., Port Coquitlam, British Columbia, Canada). The Kongsberg sonar operated at a center frequency of 200 kHz and features a user configurable pulse duration, ping rate, and source level. The average pulse duration tested was 625 µs, the ping rate was 6.9 pulses per second (pps), and the nominal source level was 195 dB re 1 µPa at 1 m. The BioSonics sonar was used in conjunction with a 210 kHz split beam digital transducer and operated at a nominal source level of 210 dB, a pulse duration of 450 µs, and a ping rate of 2.5 pps. The Imagenex sonar operated at a frequency of 260 kHz, a nominal source level of 185 dB, a pulse duration of 1000 µs, and a ping rate of 3 pps.

The characteristics of the Kongsberg sonar's transmit pulses were initially evaluated in an elongated oval laboratory tank approximately 7 m long ×3 m wide ×2 m deep. The preliminary results were presented in Deng et al. [13]. In addition to the primary peak at the sonar's 200 kHz operating frequency there is a secondary peak sound pressure level (SPL) at approximately 90 kHz. This secondary peak SPL has an amplitude of approximately 125 dB re 1 µPa at 3.5 m, which was approximately 51 dB less than the amplitude of the primary peak.

**Figure 1:**
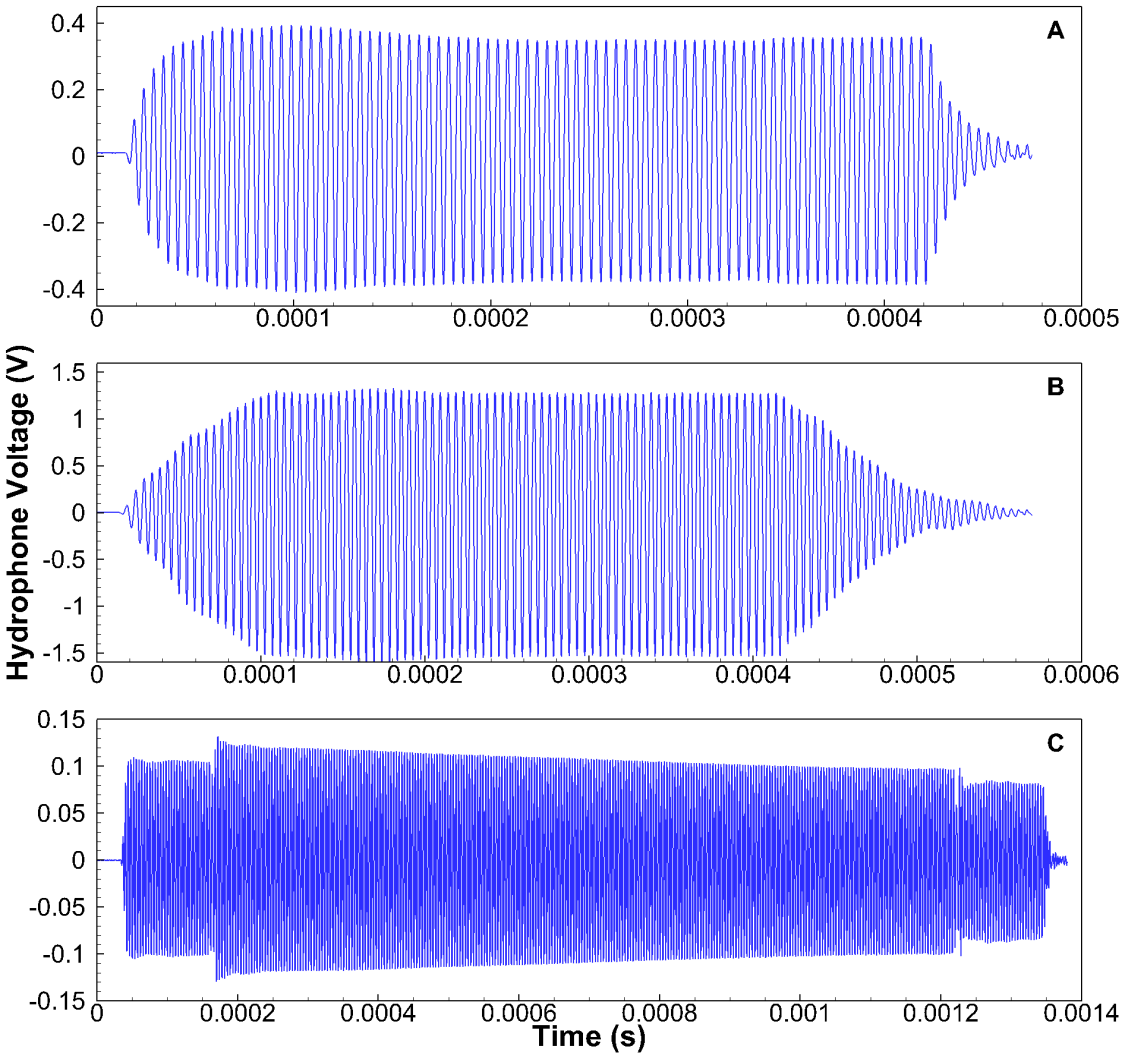
Sample raw waveforms collected for each sonar at a distance of approximately 20 m. **A**. Kongsberg (22 m); **B**. BioSonics (16 m); **C**. Imagenex (20 m).

A subsequent field evaluation of the sonars was conducted at Levey Park (46.28°N, 118.83 W) located on the Snake River at approximately river kilometer 21, where the water depth ranged from 3 m near the dock that the sonar heads were deployed from to 30 m at the furthest distance tested. The U.S. Army Corps of Engineers operates Levey Park and approved of the field testing, which did not involve endangered or protected species. The data acquisition system consisted of a calibrated TC4014-5 hydrophone (Reson Inc., Slangerup, Denmark), an EC6081 band-pass filter (Reson Inc.), a NI PXI 5922 digitizer (National Instruments [NI], Austin, Texas), a laptop computer, and the LabVIEW SignalExpress (NI) software. The hydrophone was deployed from an unpowered motorboat that was anchored at distances ranging from 7 to 200 m from the sonar head. The hydrophone has a flat (±3 dB) frequency response from 25 Hz to 250 kHz, and was tested and calibrated in an acoustic tank located in the Pacific Northwest National Laboratory Bio-Acoustics and Flow Laboratory [14], which is accredited by the American Association for Laboratory Accreditation. Each dataset was collected at a sampling rate of 10 MHz for 1 s. At least 10 datasets were collected at each distance. Each dataset was processed by isolating the individual sonar pulses and using the fast Fourier transform (FFT) method with an FFT length of 4096, the Hanning window function, and a 50% overlap for the averaging. The resulting frequency bin size was about 2.4 kHz. A sample of the waveforms collected at a distance of approximately 20 m is shown in Figure 1.

The SPL was estimated by calculating the root-mean-square of the un-weighted pressure. The estimation of the sound exposure level (SEL) is weighted by the mid-frequency cetaceans' M-weighting function given in Eq. (1) [3]:
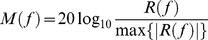
(1)where *R(f)* is given in Eq. (2),



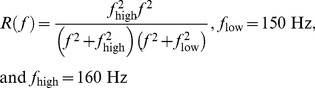
(2)A digital Butterworth band-pass filter with cutoff frequencies at 150 Hz and 160 kHz was applied to the received signal *p(t)* to obtain the M-weighted signal *p_M-mf_(t)*. The SEL was estimated in a 1 s time window using Eq. (3):
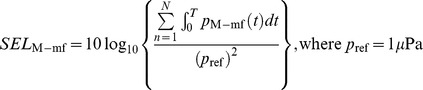
(3)


It should be noted that the analysis performed was conservative as it did not include the effects of critical ratio and temporal integration. For example it has been shown that for a 100 kHz tone the hearing threshold of a dolphin is increased by approximately 20 dB when the pulse duration is 1 ms [15].

## Results and Discussion

### 3.1 Frequency component analysis

**Figure 2:**
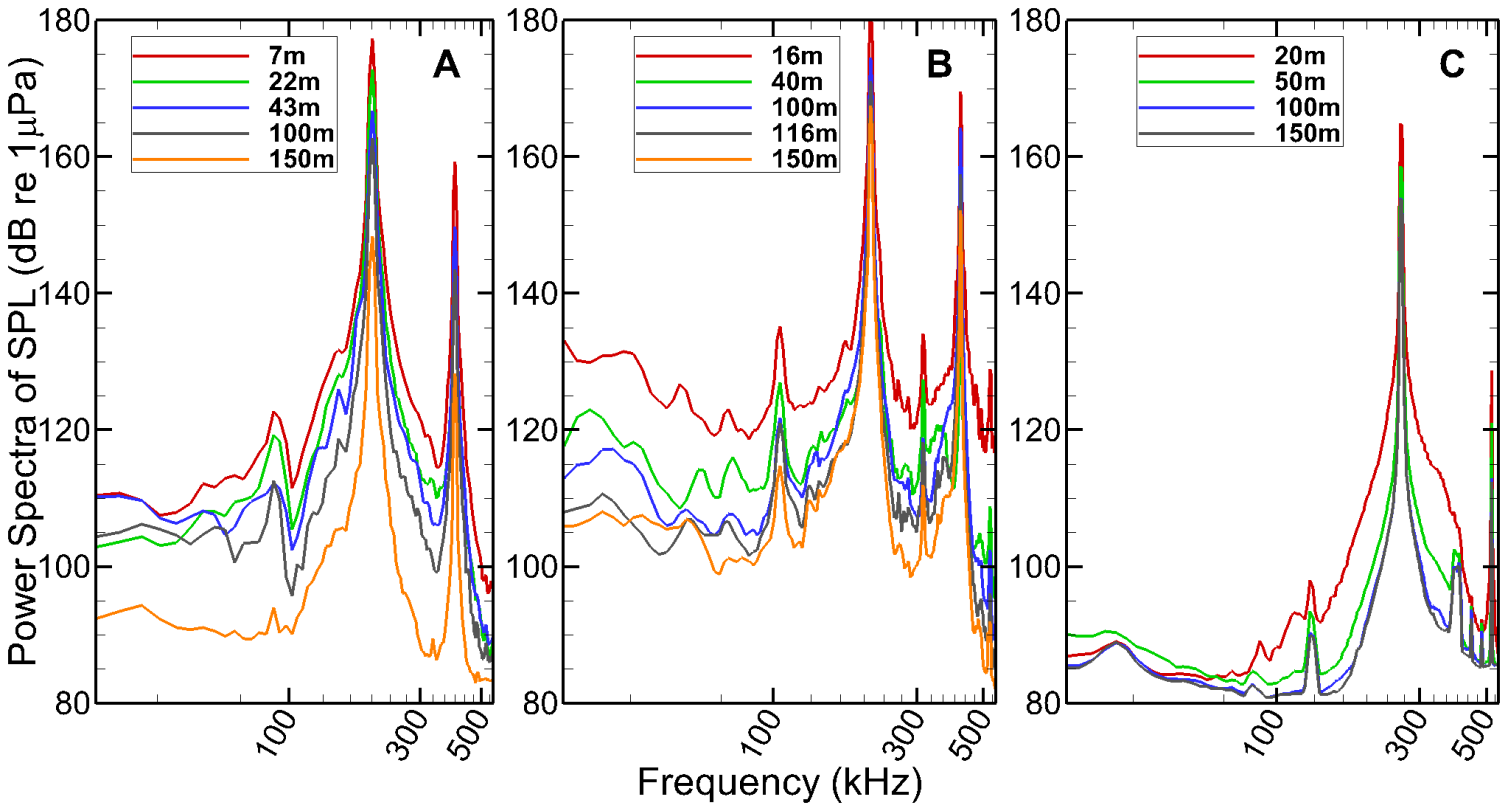
Sound pressure level spectra measured from different distances for the three sonar units. **A**. Kongsberg; **B**. BioSonics; **C**. Imagenex.

For the Kongsberg sonar, clean pulses without multipath were obtained for pulse durations up to 700 µs. Besides the main frequency of 200 kHz, there were signals at a frequency of 90 kHz with the average power from 90 to 120 dB re to 1 µPa at different distances (Figure 2A). This low frequency component stayed at 90 kHz as the pulse duration and source level were changed, and propagated through the water column and attenuated over distance similar to the main frequency component. Pure pulses without multipath were collected at all distances for the BioSonics sonar because of its narrow vertical beam angle. There was a sub-harmonic signal at 105 kHz with the average power from 100 to 130 dB re to 1 µPa (Figure 2B). For the Imagenex sonar, the pure pulse without multipath was not obtained due to its long pulse duration. There was a sub-harmonic signal at 130 kHz with the average power from 80 to 90 dB re 1 µPa (Figure 2C). There was also a peak at the sub-harmonic frequency between transmissions, although it was at least 3 dB lower than during the actual sonar pulse. It was possibly due to multiple refelctions in a relative small space.

### 3.2 Sound exposure level and sound pressure level analysis result

**Figure 3:**
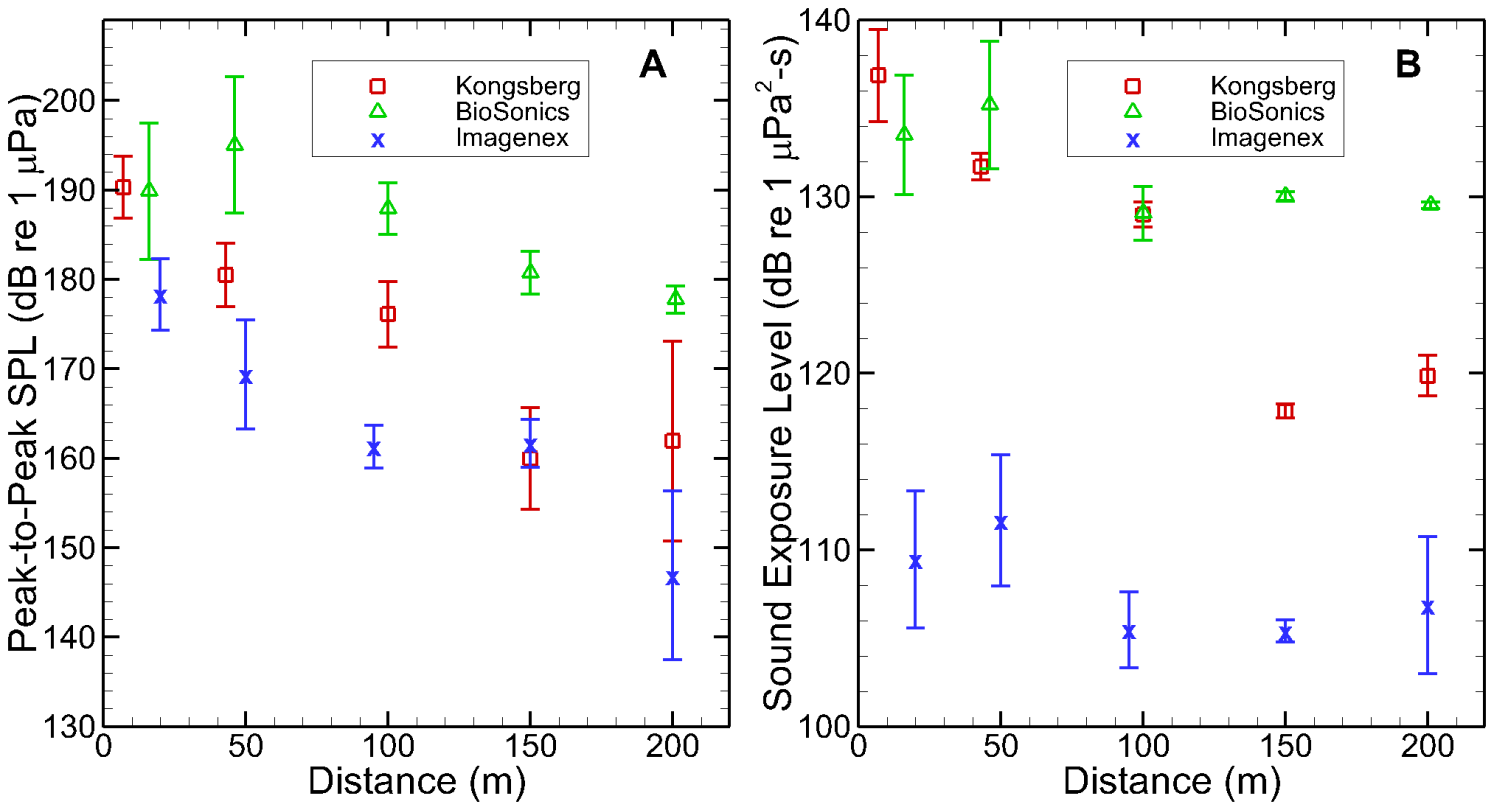
Sound pressure level and sound exposure level for three sonar units. **A**. Root-mean-square sound pressure level measurements; **B**. M_mf_ weighted sound exposure level measurements.

The root-mean-square SPL (SPL_rms_) attenuated to different degrees with range between the sonar systems. The Kongsberg sonar SPL_rms_ decreased from 181 dB to 151 dB re 1 µPa, the BioSonics sonar attenuated from 181 dB to 171 dB, and the Imagenex sonar attenuated from 171 dB to 136 dB (Figure 3A). The highest SPL_rms_ measured was 186 dB (peak SPL [SPL_pk_] of 189 dB) at a distance of 50 m for the BioSonics sonar. The M-weighted SELs from the three sonar systems (Figure 3B) decreased as distance increased. The Imagenex sonar had the lowest SEL overall. The highest SEL measured was 137 dB at 7 m away from the Kongsberg sonar.

### 3.3 Impact on killer whales

While each of these systems does generate signals at or near their specified center frequency, they also incidentally generate sounds at lower output frequencies that, while considerably lower, are clearly present and well above ambient noise levels in many operating areas. The secondary peak around 90 kHz raises the potential that some marine animals, like killer whales, false killer whales, beluga whales, Atlantic bottlenose dolphins, harbor porpoises, and others [12], may detect and be affected by these sources despite the intended operating frequency of the sonars outside the hearing range of marine mammals. Although some marine animals may detect these secondary signals the levels are below those that may cause physical harm. The measured SPLs were all below the SPL_pk_ of onset temporary threshold shift (TTS) for mid-frequency cetaceans, estimated as 224 dB in the Southall et al. criteria [3]. In addition measured SELs were all below the SEL onset TTS level of 186 dB.

While all marine mammals are protected within U.S. jurisdictions, endangered species receive a higher degree of attention, scrutiny, and protection. Within the area where many of these systems were tested and are operated, a species of key importance that is endangered is the Southern Resident Killer Whale. From personal communications with The Whale Museum at Friday Harbor during 2011, there are known situations where killer whales have been observed in the presence of these kinds of active sonar systems and were reported to be apparently detecting their presence. Based on our measurements of the incidental side-band energy generated by these nominally 200 kHz systems, killer whales are likely able to hear this secondary frequency.

It should be noted that there are other delphinids, such as Atlantic bottlenose dolphins (*Tursiops truncatus*), that have hearing thresholds that are lower at these frequencies. Based on audiograms measured by Szymanski et al. [13] using a electrophysiological method, which produces higher sensitivity estimates than behavior measures, for the secondary frequency of the Kongsberg sonar at 90 kHz the behavioral hearing threshold of killer whales is an SPL_rms_ of 70 dB re 1 µPa. The secondary frequency of the BioSonics and Imagenex sonars are slightly higher than the maximum frequency of 100 kHz from the available audiograms. As a conservative estimation of the behavioral hearing threshold of killer whales the values at 105 and 130 kHz were linearly extrapolated from the audiogram, which were 77.5 and 90 dB re 1 µPa for BioSonics and Imagenex respectively. Given these as approximate detection thresholds for signals in this frequency band, it is likely that killer whales could detect the secondary peaks of the signals.

The effective source levels of the primary and secondary frequencies were estimated for the measurements at different distances, assuming spherical spreading for the acoustic signals. The estimated effective root-mean-square source levels of the primary frequency were within 2 dB of the values given in Section 2.1, and the source levels of the secondary frequency were 141, 162, 135 dB re 1 µPa at 1 m for Kongsberg, BioSonics, and Imagenex, respectively (Table 1). The effective estimated sensation level is defined as the difference between the effective source level and the detection threshold [16]. The maximum effective estimated sensation level using behavior hearing thresholds of killer whales and the secondary frequency for each sonar unit are shown in Table 1. The resulting maximum distance over which these signals are estimated to be audible based on spherical propagation and seawater absorption [17] would be 543, 781, and 109 m for Kongsberg, BioSonics, and Imagenex respectively. For cylindrical spreading the values would be 1322, 1540, and 460 m for Kongsberg, BioSonics, and Imagenex respectively. The actual distance that these signals will be audible will vary with the sea state (noise and propagation changes) and bathymetry. The propagation model will likely be between the spherical and cylindrical estimates.

**Table 1:** Estimated effective root-mean-square source level of the secondary frequency and the resulting distance over which these signals would be audible assuming spherical spreading in seawater (values assuming cylindrical spreading shown in parenthesis).

Sonar	Operating frequency (kHz)	Frequency of secondary peak (kHz)	Source level of secondary peak (dB re 1 µPa at 1 m)	Hearing Threshold (dB re 1 µPa)	Sensation level (dB re 1 µPa)	Audible range (m)
Kongsberg	200	90	141	70	71	543 (1322)
BioSonics	210	105	162	77.5	84.5	781 (1540)
Imagenex	260	130	135	90	45	109 (460)

Based on these calculations from measured signals in the field and controlled conditions and what is known about the hearing in killer whales, it appears likely that they may detect the incidental side-lobe energy of these signals over ranges of several hundred meters. Consequently, their behavior could be potentially affected by the presence of these systems in the close proximity to them. Responses could include attraction out of interest in the presence of a signal and what it may indicate or they could induce an avoidance response, which in the case of operational energy-producing devices with moving parts might not necessarily be an undesirable result. Based on all the evidence available for the effects of noise on hearing in marine mammals, estimated noise exposure criteria for impulsive sounds on cetaceans [3], these sounds are almost certainly not directly harmful to the hearing of killer whales or other marine mammals even directly at the source. In addition, it has been reported that killer whales have avoided narrow-band acoustic signals at moderate levels [3], [18]. Therefore, any potential impact issues seem to be related to detection and behavioral response rather than direct injury from the lower frequency component of these sounds. Nevertheless, the existence of considerable energy at lower frequencies within the functional hearing range of killer whales and other odontocetes, raises the issue of whether such systems should be flatly excluded from consideration in impact assessment based solely on their supposed nominal operating frequency.

## Conclusions

Measurements of the spectral properties of sound pulses transmitted by three commercially available 200 kHz echo sounders under typical operation conditions are consistent with some observations of the behavioral response of some marine mammals exposed to such transmissions that the sounders were generating sound within the hearing range of the animals. While on the order of 50 dB down in amplitude from the sounders' center frequencies, the level of sound within the hearing range of some marine mammals was found to be above the thresholds for hearing of many marine mammals but well below the levels that might cause physical injury. The range at which the side lobe sound generated by active acoustic devices operating near 200 kHz might be heard by marine mammals will be a function of the characteristics of the sounder and the level and spectrum of natural and anthropogenic noise that occurs during the time the sonar may be operated. Regulatory authorities may want to reconsider the unquestioned exclusion of echo sounders from consideration of environmental impact based entirely on their primary/center operating frequency.

## References

[1] NRC (National Research Council) (2003) Ocean noise and marine mammals. Washington DC: National Academies Press. 617p. 25057640

[2] Hildebrand JA (2009) Anthropogenic and natural sources of ambient noise in the ocean. Mar Ecol Prog Ser 395: 5–20 10.3354/meps08353

[3] Southall BL, Bowles AE, Ellison WT, Finneran JJ, Gentry RL, et al (2007) Marine mammal noise exposure criteria: initial scientific recommendations. Aquatic Mammals 33(4): 411–522 10.1578/AM.33.4.2007.411

[4] Melcón ML, Cummins AJ, Kerosky SM, Roche LK, Wiggins SM, et al (2012) Blue Whales Respond to Anthropogenic Noise. PLoS ONE 7(2): e32681 10.1371/journal.pone.0032681 22393434 PMC3290562

[5] Pirotta E, Milor R, Quick N, Moretti D, Di Marzio N, et al (2012) Vessel Noise Affects Beaked Whale Behavior: Results of a Dedicated Acoustic Response Study. PLoS ONE 7(8): e42535 10.1371/journal.pone.0042535 22880022 PMC3411812

[6] Tyack PL, Zimmer WMX, Moretti D, Southall BL, Claridge DE, et al (2011) Beaked Whales Respond to Simulated and Actual Navy Sonar. PLoS ONE 6(3): e17009 10.1371/journal.pone.0017009 21423729 PMC3056662

[7] Solé M, Lenoir M, Durfort M, López-Bejar M, Lombarte A, et al (2013) Ultrastructural Damage of Loligo vulgaris and Illex coindetii statocysts after Low Frequency Sound Exposure. PLOS ONE 8(10): e78825 10.1371/journal.pone.0078825 24143265 PMC3797068

[8] Popper AN, Hastings MC (2009) The effects of human-generated sound on fish. Interactive Zoology 4(1): 43–52 10.1111/j.17494877.2008.00134.x 21392276

[9] Li S, Nachtigall PE, Breese M, Supin AY (2012) Hearing Sensation Levels of Emitted Biosonar Clicks in an Echolocating Atlantic Bottlenose Dolphin. PLOS ONE 7(1): e29793 10.1371/journal.pone.0029793 22238654 PMC3253102

[10] Deng ZD, Carlson TJ, Fu T, Ren H, Martinez JJ, et al (2013) Design and Implementation of a Marine Animal Alert System to Support Marine Renewable Energy. Marine Technology Society Journal 47(4): 113–121 10.4031/MTSJ.47.4.2

[11] Au WWL, Hastings MC (2008) Principles of Marine Bioacoustics. New York: Springer. 617p. doi:10.1007/978-0-387-78365-9

[12] Szymanski MD, Bain DE, Kiehl K, Pennington S, Wong S, et al (1999) Killer whale (Orcinus orca) hearing: Auditory brainstem response and behavioral audiograms. Journal of the Acoustical Society of America 106: 1134–1141 10.1121/1.427121 10462816

[13] Deng ZD, Carlson TJ, Xu JS, Martinez JJ, Weiland MA, et al. (2011) Design and Operation Specifications of an Active Monitoring System for Detecting Southern Resident Killer Whales. Oceans 2011 Kona, Hawaii 19–22 September 2011.

[14] Deng ZD, Weiland MA, Carlson TJ, Eppard MB (2010) Design and Instrumentation of a Measurement and Calibration System for an Acoustic Telemetry System. Sensors 10: , 3090–3099. doi:10.3390/s100403090 10.3390/s100403090 PMC3274180 22319288

[15] Johnson CS (1968) Relationship Between Absolute Threshold and Duration of Tones Pulse in the Bottlenosed Porpoise. Journal of the Acoustical Society of America 44: , 757–763. doi:10.1121/1.1910893 10.1121/1.1910893 5645824

[16] Ellison WT, Southall BL, Clark CW, Frankel AS (2012) A New Context-Based Approach to Assess Marine Mammal Behavioral Responses to Anthropogenic Sounds. Conservation Biology 26: 21–28 10.1111/j.15231739.2011.01803.x 22182143

[17] Ainslie MA, McColm JG (1998) A simplified formula for viscous and chemical absorption in sea water. Journal of the Acoustical Society of America 103: 1671–1672 10.1121/1.421258

[18] Miller PJO, Atunes RN, Wensveen PJ, Samarra FIP, Alves AC, et al (2014) Dose-response relationships for the onset of avoidance of sonar by free-ranging killer whales. Journal of the Acoustical Society of America 135(2): 975–993 10.1121/1.4861346 25234905

